# Retraction: Efficient *in vivo* wound healing using noble metal nanoclusters

**DOI:** 10.1039/d2nr90041f

**Published:** 2022-03-10

**Authors:** Kuo Li, Dan Li, Cheng-Hsuan Li, Pengfei Zhuang, Chunmei Dai, Xiangka Hu, Dahao Wang, Yuanye Liu, Xifan Mei, Vincent M. Rotello

**Affiliations:** a Department of Orthopedics, The First Affiliated Hospital of Jinzhou Medical University, 40 Songpo Road, Jinzhou, China.; b Department of Chemistry, University of Massachusetts Amherst, 710 North Pleasant Street, Amherst, Massachusetts 01003, USA.

We, the named authors, hereby wholly retract this *Nanoscale* article due to errors in the labelling and filing of the data, which has led to ambiguity over the correct data for each figure.

In Fig. 1a, there is overlap between the 1h control panel and the 1h GSH-AgNCs panel.

In Fig. 5, there is overlap between the H&E 200× control panel and the H&E 200× GSH-AgNCs panel, and between the H&E 200× DHLA-AgNCs panel and the H&E 200× DHLA-AuNCs panel.

In Fig. S12, there is overlap between all of the panels for the control and the DHLA-AgNCs.

The original data is available, but the order and naming of figures are not reliable. Since the other figures still support the conclusion, the data related to Fig. 1a, 5, and Fig. S12 will be redone and verified multiple times.

Signed: Kuo Li, Dan Li, Cheng-Hsuan Li, Pengfei Zhuang, Chunmei Dai, Xiangka Hu, Dahao Wang, Yuanye Liu, Xifan Mei and Vincent M. Rotello

Date: 16/2/2022

Retraction endorsed by Heather Montgomery, Managing Editor, *Nanoscale*

